# New Works

**Published:** 1859-09

**Authors:** 


NEW WORKS.—Messrs. Blanchard & Lea have in the press, and will issue early in the autumn, Professor Austin Flint's treatise on the Diseases of the Heart; Professor Hamilton's work on Fractures; and Professor Stillé's treatise on Materia Medica and Therapeutics—the latter in two large octavo volumes. Dr. Da Costa's work on Diagnosis is in the press of Messrs. J. B. Lippincott & Co., and will appear in the course of the winter.

